# Superhydrophobic Water-Solid Contact Triboelectric Generator by Simple Spray-On Fabrication Method

**DOI:** 10.3390/mi9110593

**Published:** 2018-11-13

**Authors:** Jihoon Chung, Deokjae Heo, Banseok Kim, Sangmin Lee

**Affiliations:** School of Mechanical Engineering, Chung-Ang University, 84, Heukseok-ro, Dongjak-gu, Seoul 06974, Korea; jihoon@cau.ac.kr (J.C.); ejrwo472@naver.com (D.H.); show8910@naver.com (B.K.)

**Keywords:** energy harvesting, triboelectric nanogenerator, superhydrophobic surface, spray method, mechanical energy

## Abstract

Energy harvesting is a method of converting energy from ambient environment into useful electrical energy. Due to the increasing number of sensors and personal electronics, energy harvesting technologies from various sources are gaining attention. Among energy-harvesting technologies, triboelectric nanogenerator (TENG) was introduced as a device that can effectively generate electricity from mechanical motions by contact-electrification. Particularly, liquid-solid contact TENGs, which use the liquid itself as a triboelectric material, can overcome the inevitable friction wear between two solid materials. Using a commercial aerosol hydrophobic spray, liquid-solid contact TENGs, with a superhydrophobic surface (contact angle over 160°) can be easily fabricated with only a few coating processes. To optimize the fabrication process, the open-circuit voltage of sprayed superhydrophobic surfaces was measured depending on the number of coating processes. To demonstrate the simple fabrication and applicability of this technique on random 3D surfaces, a liquid-solid contact TENG was fabricated on the brim of a cap (its complicated surface structure is due to the knitted strings). This simple sprayed-on superhydrophobic surface can be a possible solution for liquid-solid contact TENGs to be mass produced and commercialized in the future.

## 1. Introduction

Energy harvesting is a method of converting energy from ambient environment into useful electrical energy. Harvesting energy from the ambient environment is gaining more and more interest due to the increasing number of sensors inside personal electronic devices, which consume extra power and drain batteries much faster. In this respect, there is an increasing number of studies on the use of solar [1], thermal [2] and RF [3] energy to power various sensors and electrical components. Among these energy sources, mechanical energy is one of the desirable sources that is not affected by external environment such as weather, location and so forth. To produce electricity from a mechanical input, piezoelectric [4,5], capacitive [6] and electromagnetic transduction [7] generators have been developed. Recently, the triboelectric nanogenerator (TENG) was introduced as a solution that can effectively generate electricity from mechanical motions by contact electrification [8,9,10,11,12]. In particular, liquid-solid contact TENGs, which use the liquid itself as a triboelectric material, are in the spotlight because they can overcome the inevitable friction wear between two solid materials in conventional TENGs [13,14,15]. For liquid-solid contact TENGs to produce a sustainable energy output, the solid surface must be superhydrophobic to repel the liquid after it falls. However, producing a superhydrophobic coating on metal or polymer surfaces requires complex fabrication procedures such as vapor deposition [16], plasma treatment [17], or self-assembled monolayer coating [18] to create micro-/nanostructures and to lower the surface energy. In addition, these methods have limited applications for complex 3D surfaces. Therefore, for liquid-solid contact TENGs to be commercialized, the fabrication procedures need to be simple and appropriate for mass production.

In this work, we introduce a liquid-solid contact TENG with a superhydrophobic surface fabricated through a spray-on method; this method can also simply produce a superhydrophobic coating on a complex 3D surface. With a commercial aerosol spray, a superhydrophobic surface that has contact angle of over 160° is created just after few coatings. With a simple superhydrophobic surface, the sprayed-on TENG can generate about 30 V per water drop. To optimize the fabrication process, the open-circuit voltage of the sprayed superhydrophobic surface depending on the number of coating process was measured. The sprayed superhydrophobic coating was shown to be maintained even after being subjected to 20 h of water drops (every 0.5 s). The sprayed superhydrophobic surface was able to produce an average peak open-circuit voltage (*V_OC_*) of 13.4 V and a closed-circuit current (*I_CC_*) of 2.1 μA under continuous water spraying from a commercial shower head. To demonstrate the simple fabrication and applicability on a random 3D surface, a liquid-solid contact TENG was fabricated on the brim of a cap, which has a complicated surface structure due to the knitted strings. The sprayed-on TENG cap produced sufficient electrical output to light up an LED. The superhydrophobic surface created by the aerosol spray method presented in this study can be easily applied to 3D surfaces using a simple fabrication process. Thus, this technique can be a possible solution for liquid-solid contact TENGs to be mass produced in the future.

## 2. Materials and Methods

### 2.1. The Fabrication Process of Sprayed-On Superhydrophobic Surface

First, a 10 cm × 10 cm bare aluminum plate was cleaned with ethyl alcohol and deionized water (DI-water). Then, the base-coat spray was applied on the bare aluminum plate and dried for 1 min. This procedure was repeated three times and the surface was dried for 30 min to obtain a well-coated adhesive layer. Next, the top-coat spray was applied on top of the adhesive layer and dried for 1 min. This process was repeated several times and the surface was dried for 12 h to fix the hydrophobic layer onto the adhesive layer. For the sprayed-on TENG cap, cleaning process with ethyl alcohol and deionized water was omitted.

### 2.2. Contact Angle Measurement Methods

The contact angle of each surface was measured with a drop-shape analysis device (SmartDrop, Femtofab Co., Seongnam, Korea); the average was determined from five measurements at different locations for each surface.

## 3. Results and Discussion

**Figure 1:**
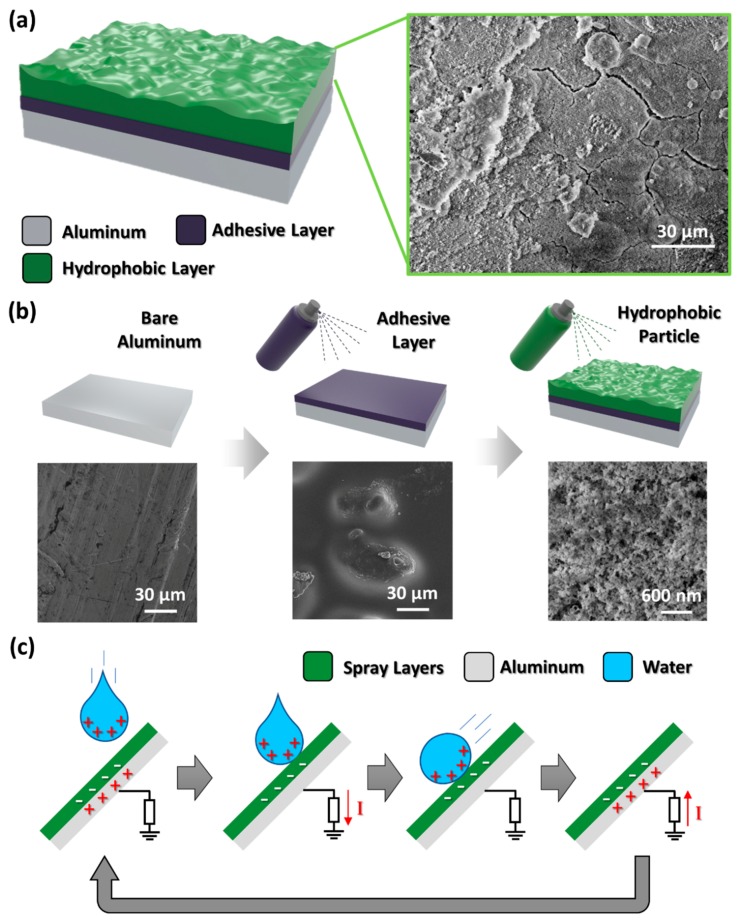
(**a**) Schematic illustration of sprayed-on superhydrophobic surface and field-emission scanning electron microscopy (FE-SEM) image; (**b**) Fabrication method of sprayed-on superhydrophobic surface and corresponding FE-SEM images; (**c**) Working mechanism of sprayed-on TENG.

The sprayed-on TENG is composed of three layers: an aluminum layer as the base substrate, an adhesive layer in the middle and a hydrophobic layer on the top (Figure 1a). The field-emission scanning electron microscopy (FE-SEM) image on the right shows the top view of the sprayed hydrophobic layer, in which the scale length is 30 μm. As shown in the FE-SEM image, the polymer layer is well-established after a simple spray coating process. Figure 1b shows the entire fabrication process using a commercial hydrophobic spray (NeverWet, RUST-OLEUM). The magnified images below the fabrication schematics are FE-SEM images taken from samples during each process. As shown in Figure 1a, the aluminum surface does not have any micro/nano structures on the surface. As shown in second FE-SEM image (Figure 1b), a sticky polymer layer was formed on the surface of the aluminum substrate. In the third FE-SEM image (Figure 1b, with a scale of 600 nm), hydrophobic nanoparticles were spread and fixed on the adhesive layer and the actual surface area of the top surface increased. According to Wenzel’s equations [19], the roughness factor (the ratio between the actual surface and the geometric surface) increases as nanostructures are fabricated on the aluminum substrate and the surface becomes superhydrophobic. Additional FE-SEM images are provided in Supplementary Materials Figure S1, where Figure S1a–d represent the bare aluminum surface, the adhesive layer surface, the hydrophobic surface with the top coat and the magnified hydrophobic surface with the top coat, respectively.

The working mechanism of the sprayed-on TENG is based on the single-electrode-mode TENG, a liquid-solid contact TENG studied in previous works [14,20]. In Figure 1c, the water droplet is positively pre-charged due to various environmental factors such as friction with air or pipe [13]. And sprayed superhydrophobic surface is negatively pre-charged because water is preliminarily dropped several times and accordingly has triboelectric interactions with the surface. When the water droplet falls onto the sprayed superhydrophobic layer, an electric potential equilibrium is formed, causing current to flow instantaneously from the electrode (aluminum) to the ground. When the water droplet is in complete contact with the superhydrophobic surface, the electrode becomes neutral and no current flows. When the water droplet naturally slides down due to gravity, current reflows instantly from the ground to aluminum layer due to electrostatic induction by the charged hydrophobic layer. This working process is repeated for each water droplet that falls onto the superhydrophobic surface.

**Figure 2:**
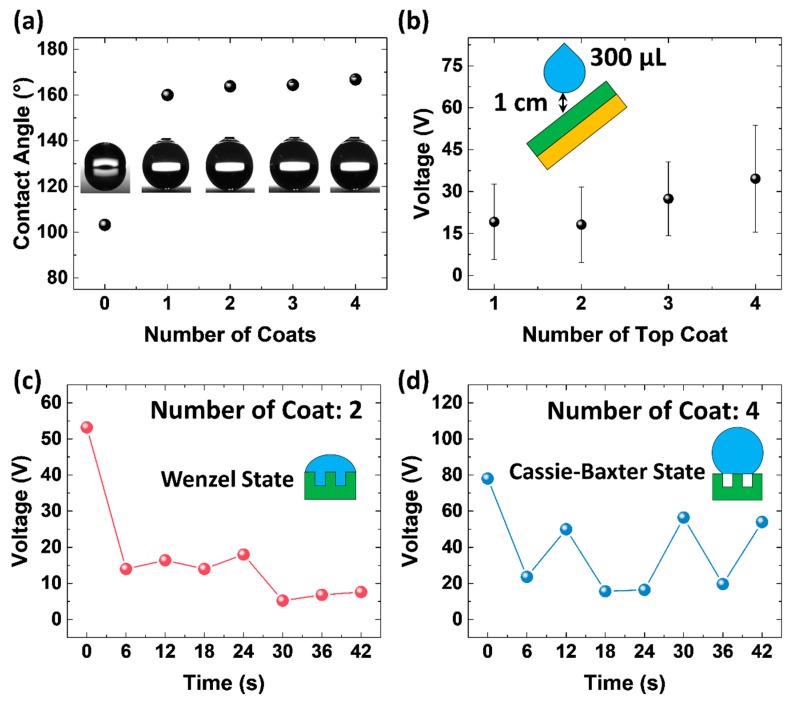
(**a**) Contact angle and (**b**) *V_OC_* output depending on the number of top coats applied. Peak *V_OC_* value of sprayed-on triboelectric nanogenerator (TENG) with (**c**) 2 top coats and (**d**) 4 top coats.

Figure 2a shows the contact angle of the sprayed superhydrophobic surface as a function of the number of coats. As shown in the plot, the bare aluminum used in this study showed an average contact angle of 103.2° when 3 μL of DI-water was dropped on the surface. After the first layer of the top coat was applied, the average contact angle increased to 160°. As the number of top coats increased, the contact angle increased slightly. 

However, as shown in Figure 2b, the *V_OC_* output of the sprayed-on TENG changed dramatically depending on the number of top coats applied on the aluminum surface. The *V_OC_* output was measured when 300 μL of tap water was dropped (1 drop every 6 s) from a height of 1 cm onto the hydrophobic surface. The sprayed-on TENG was tilted approximately 60° to the ground for the water drops to be separated naturally after coming into contact with the TENG surface. With a single top coat, the average *V_OC_* output was 19.1 V, which increases to 34.6 V when 4 coats were applied to the TENG surface. This is because the ability of the sprayed hydrophobic surface to withstand the hydraulic pressure of the water drops varies depending on the number of top coats. For a super-hydrophobic surface to be sustained drop after drop, air pockets in between micro-/nanostructures are necessary; this is to ensure that the water drops are in the Cassie-Baxter state. A liquid-solid contact is in the Cassie-Baxter state when two criteria are met: (i) the perimeter of the surface structures are greater than the body forces and (ii) the surface structures are taller than the liquid protruding between them, so that the liquid does not come into contact with the base of the solid [21]. The contact angle of the sprayed superhydrophobic surface using 3 μL of DI-water may show super-hydrophobicity (Figure 2a) but as the hydraulic pressure is increased when using 300 μL of tap water falling from certain height, the liquid can penetrate through the surface structures and come into contact with the base surface. This causes the air pockets in between the micro-/nanostructures to be filled with water (Wenzel state) and the surface is no longer superhydrophobic.

The experimental result is shown in Supplementary Material Figure S2, where the water drops remained on the sprayed hydrophobic surface with 1~2 top coats. The sprayed hydrophobic surface with 3~4 top coats remained dry even after 300 μL of tap water was applied onto the surface. The *V_OC_* output of each surface shows same results as well. Figure 2c,d are plots of the peak *V_OC_* values when 300 μL of tap water was applied, with 1 drop every 6 s. As shown in Figure 2c, the initial voltage with 2 top coats was 53.2 V, which then decreased drastically. When the first drop of water falls onto the surface, the surface is completely dry, so the sprayed-on TENG can produce a high output. However, the first drop forms a Wenzel state with the solid surface and the water drop is pinned onto the surface. The water remaining from the first drop interferes with the second drop and decreases the electric potential between this second drop and the solid surface [20]. As a result, the *V_OC_* output of the sprayed-on TENG decreases after the first drop. In contrast, the water drop forms a Cassie-Baxter state with the superhydrophobic surface with 4 top coats; thus, the water drop rolls off immediately after coming into contact with the solid surface, leaving only a small amount of or no water residue (Figure 2d). As shown in the plot, the peak *V_OC_* output decreases after the first drop, similar to the sprayed-on TENG with 2 coats. However, the remaining water residue is small, so it can evaporate or become detached easily from the surface; this results in less interference between the next drop and the solid surface. The experimental results in Figure 2d also shows recovered *V_OC_* output during the experiment. Therefore, on the average, the sprayed-on TENG with 4 top coats has a higher output than the sprayed-on TENG with 2 top coats.

**Figure 3:**
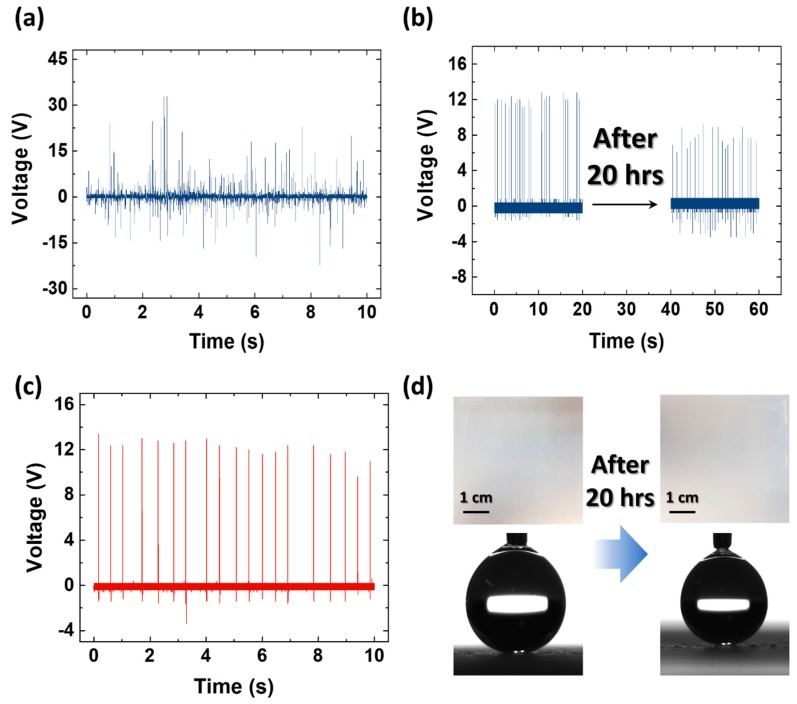
(**a**) *V_OC_* output of sprayed-on TENG with water applied using a commercial shower head; (**b**) *V_OC_* output of sprayed-on TENG after 20 h of water application; (**c**) Recovered *V_OC_* output of sprayed-on TENG after drying; (**d**) Surface and contact angle of sprayed-on TENG before and after 20 h of water application.

Figure 3a shows the continuous *V_OC_* output of the sprayed-on TENG with 4 top coats when tap water was sprayed continuously with a shower head. The average positive peak *V_OC_* output is 13.4 V. The continuous *I_CC_* output is shown in Supplementary Material Figure S3, where the average positive peak *I_CC_* output is measured to be 2.1 μA. Also, each corresponding magnified voltage and current graph is shown in Supplementary Material Figure S4. As the lifetime of a sprayed superhydrophobic surface is important, the sprayed-on TENG with 4 top coats was exposed to a continuous water-drop condition using tap water (Figure 3b). Single commercial tap water was dropped every 0.5 s for 20 h. As shown in the plot, the sprayed-on TENG initially produced a peak *V_OC_* of about 12 V but after 20 h, the *V_OC_* output decreased to 7 V. However, the decreased output was due to the water residue on the superhydrophobic surface. After drying for 3 h, the sprayed-on TENG showed the same *V_OC_* value as the initial output (Figure 3c). As shown in Figure 3d, the surfaces before and after 20 h of water application showed no differences when observed with the naked eye; in addition, the contact angle of the superhydrophobic surface remained the same (over 160°). This result indicates that surface structure of the sprayed-on TENG remains the same after the experiment. 

**Figure 4:**
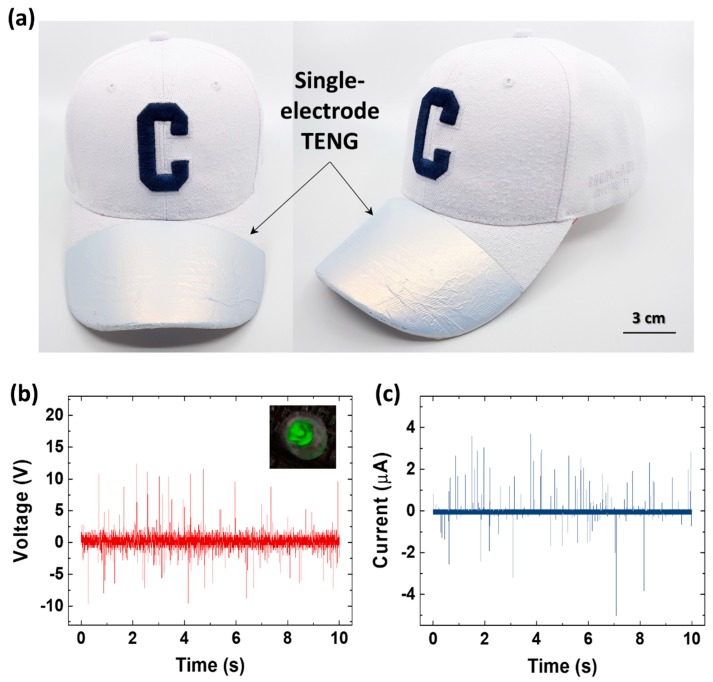
(**a**) Photograph of sprayed-on TENG cap. (**b**) *V_OC_* and (**c**) *I_CC_* output of sprayed-on TENG cap when water was applied using a commercial shower head.

With a spray-on method, a liquid-solid contact TENG can be created on any complex 3D structure. In this work, a liquid-solid contact TENG was fabricated on the brim of a cap, which has a complicated surface structure due to the knitted yarns. Overall, the fabrication process is the same as the process used for the sprayed-on TENG in Figure 1b. Aluminum foil (electrode) was attached to the brim of the cap instead of an aluminum plate. In the sprayed-on TENG cap, the aluminum foil on the brim and all other parts of the cap was coated. Even though only the aluminum foil works as an electrode, both the cap fabric and the aluminum electrode need to be superhydrophobic; this is because if the cap fabric is even partially wet, water molecules can propagate through the yarns and a large area of the fabric gets wet eventually. If the fabric directly in contact with the aluminum foil gets wet, the electrical potential between the falling water drop and the aluminum electrode is significantly reduced, resulting in less or no electrical output. Figure 4a shows an actual photograph of a sprayed-on TENG cap, which is similar to a single-electrode-mode TENG. As shown in the photograph, both the aluminum foil and the cap fabric were coated with the hydrophobic spray. 

The fabricated sprayed-on TENG cap was pre-tested and checked for super-hydrophobicity by using tap water sprinkled with a commercial shower head (Supplementary Material Figure S5, Supplementary Video S1). As shown in the photographs, the sprayed-on TENG cap repelled all the water drops from the shower head and there were no water drops left on the surface. Figure 4b,c represent the continuous *V_OC_* and *I_CC_* outputs of the sprayed-on TENG cap, respectively, during the experiment. The electrical output of the sprayed-on TENG cap was sufficient to light up a LED when connected to a rectifier circuit, as shown in the inset of Figure 4b. Detailed circuit is shown in Supplementary Material Figure S6. In addition, to show a stability of sprayed superhydrophobic coating visually, bending test was conducted on paper, polyimide, polyurethane film sample. First, each 2 cm × 5 cm sample followed same fabrication process used for the sprayed-on TENG in Figure 1b and was checked for super-hydrophobicity by DI-water droplets (Supplementary Video S2). Next, both ends of each sample (about 1 cm) was attached on plates of commercial vibration tester (ET-126B-4, Labworks Co., Costa Mesa, CA, USA) and bending was applied two thousand times by constant amplitude (about 1 cm) of vibration, as shown in Supplementary Video S3. Finally, each sample was successfully checked for super-hydrophobicity by DI-water (Supplementary Video S4). This result demonstrated sprayed superhydrophobic coating has infinite potential to be utilized as TENG by applying all kinds of materials.

## 4. Conclusions

In summary, we developed a sprayed-on TENG using a commercial hydrophobic spray that can easily create a superhydrophobic surface. The surface has a contact angle of over 160°, which was achieved with only a few spraying processes. The electrical output depends on the number of top coats applied on the solid surface; it was determined that the electrical output could be maximized by maintaining a Cassie-Baxter state between the water drop and the superhydrophobic surface. The sprayed-on superhydrophobic surface produced an average positive peak *V_OC_* of 13.4 V and *I_CC_* of 2.1 μA under continuous water sprinkling from a commercial shower head. The sprayed-on superhydrophobic surface was able to withstand 20 h of water drops falling every 0.5 s from a tap without surface damage. To demonstrate the easy application of the spray-on method on a complex 3D surface, a superhydrophobic surface was created on the brim of a cap. The sprayed-on TENG cap was able to light up a LED when water was applied. Therefore, this simple spray-on method to create a superhydrophobic surface can be a potential solution for mass-production and commercialization of liquid-solid contact TENGs in the future.

## Supplementary Materials

The following are available online at http://www.mdpi.com/2072-666X/9/11/593/s1, Figure S1: Additional FE-SEM Images, Figure S2: Photographs of superhydrophobic surfaces after dropping 300 μL of water, Figure S3: *I_CC_* output of sprayed-on TENG when water was applied with a commercial shower head, Figure S4: Magnified *V_OC_* and *I_CC_* output of sprayed-on TENG when water was applied with a commercial shower head, Figure S5: Photographs of sprayed-on TENG cap when water is applied with a commercial shower head, Figure S6: Sprayed-on TENG connected to a circuit to light an LED, Video S1: Water applied onto the sprayed-on TENG cap when with a commercial shower head, Video S2: DI-water applied onto the paper, polyimide, polyurethane film sample before bending test, Video S3: Bending test with a commercial vibration tester, Video S4: DI-water applied onto the paper, polyimide, polyurethane film sample after bending test.
