# Supplementary material for: Superhydrophobic Water-Solid Contact Triboelectric Generator by Simple Spray-On Fabrication Method
Source: Micromachines (Basel). 2018 Nov 13;9(11):593. doi: 10.3390/mi9110593 (PMC6266004; doi:10.3390/mi9110593)
Supplement: Supplementary file 1 [file micromachines-09-00593-s001.zip › micromachines-387052-supplementary/micromachines-387052-supplementary.docx]

Supplementary Materials: Superhydrophobic Water-Solid Contact Triboelectric Generator by Simple Spray-On Fabrication Method


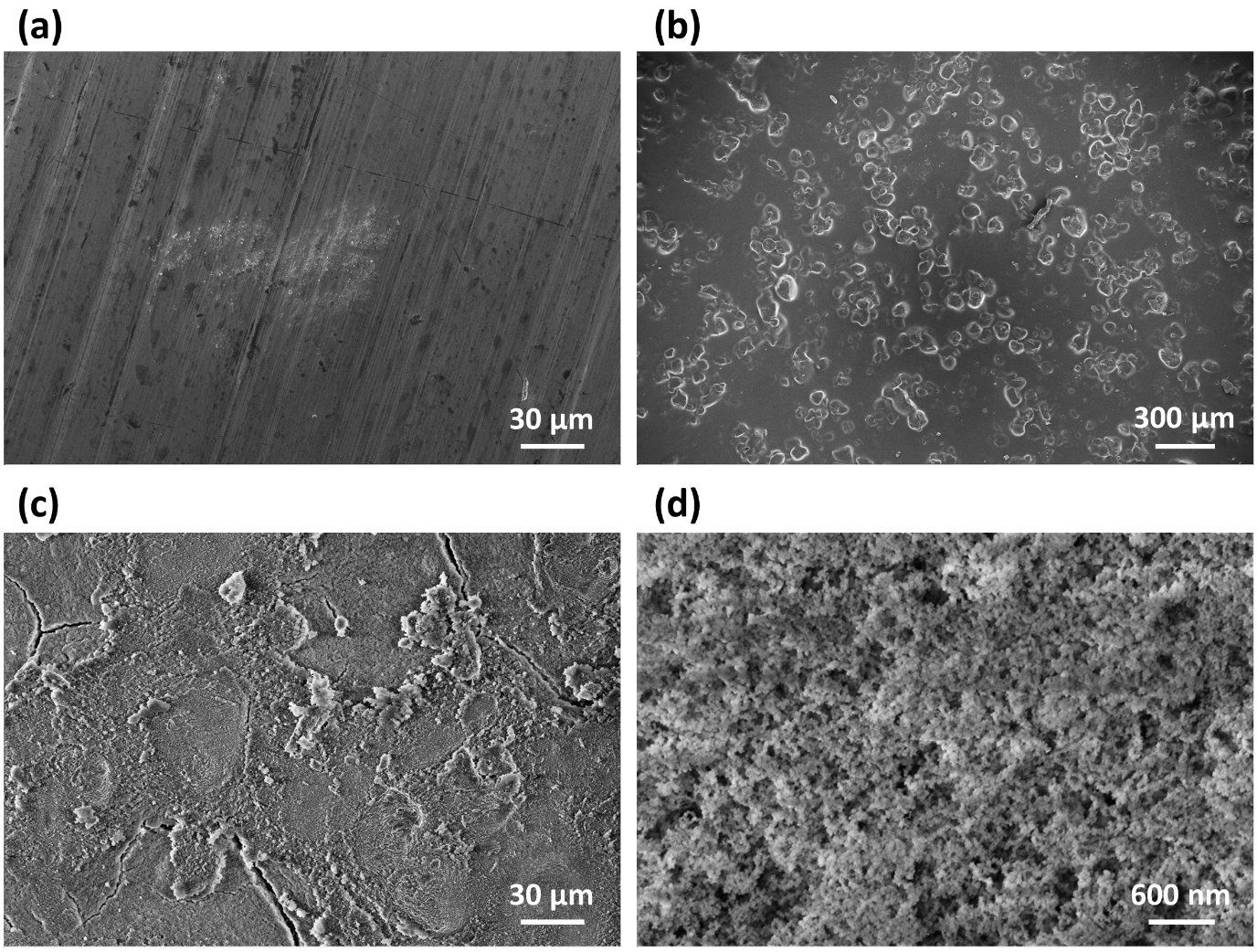


**Figure S1.** FE-SEM image of (**a**) aluminum, (**b**) the adhesive layer, and (**c**) the superhydrophobic layer. (**d**) Magnified FE-SEM image of the superhydrophobic layer.


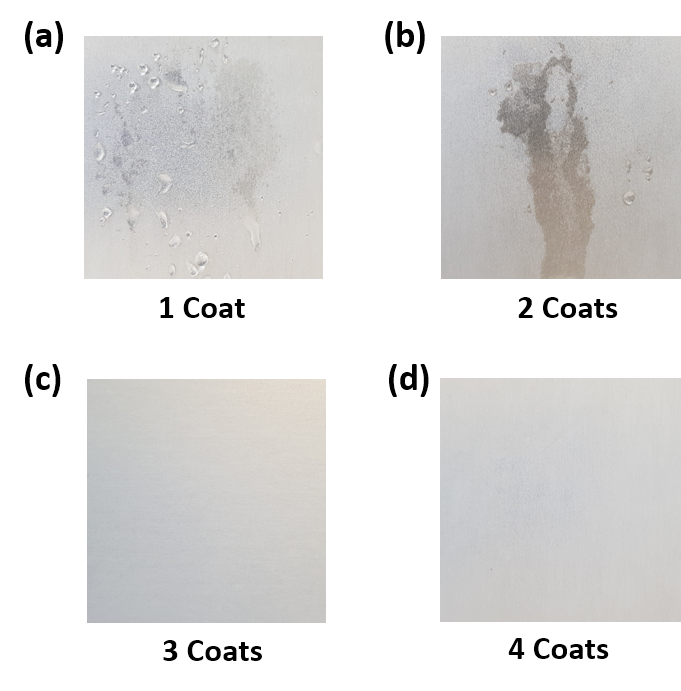


**Figure S2.** Photographs of superhydrophobic surfaces after dropping 300 μL of water: (**a**) 1 top coat, (**b**) 2 top coats, (**c**) 3 top coats, (**d**) 4 top coats.


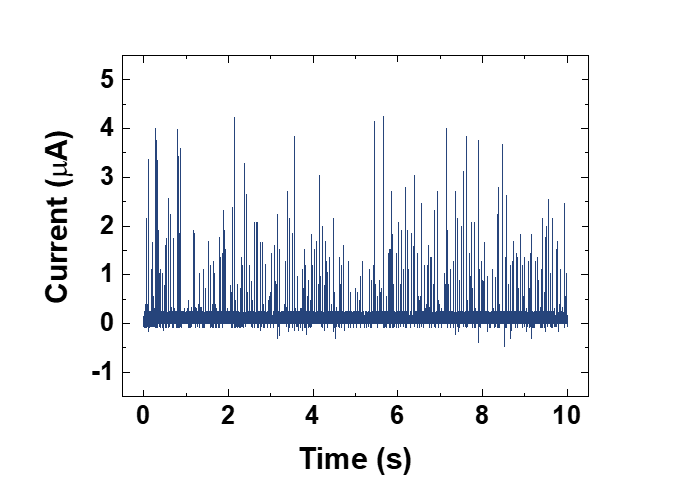


**Figure S3.** *I_CC_* output of sprayed-on TENG when water was applied with a commercial shower head.


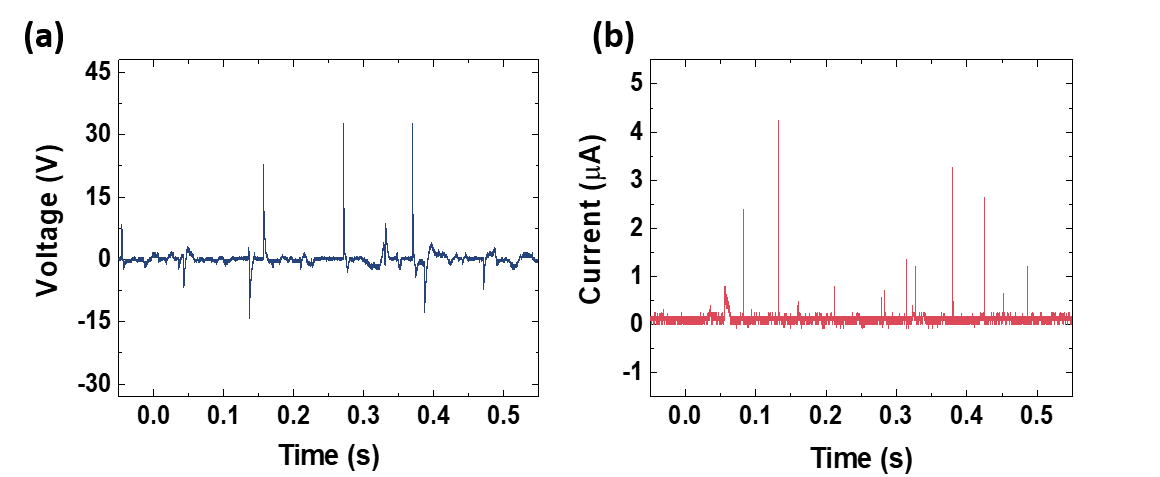


**Figure S4.** Magnified (**a**) *V_OC_* and (**b**) *I_CC_* output of sprayed-on TENG when water was applied with a commercial shower head.


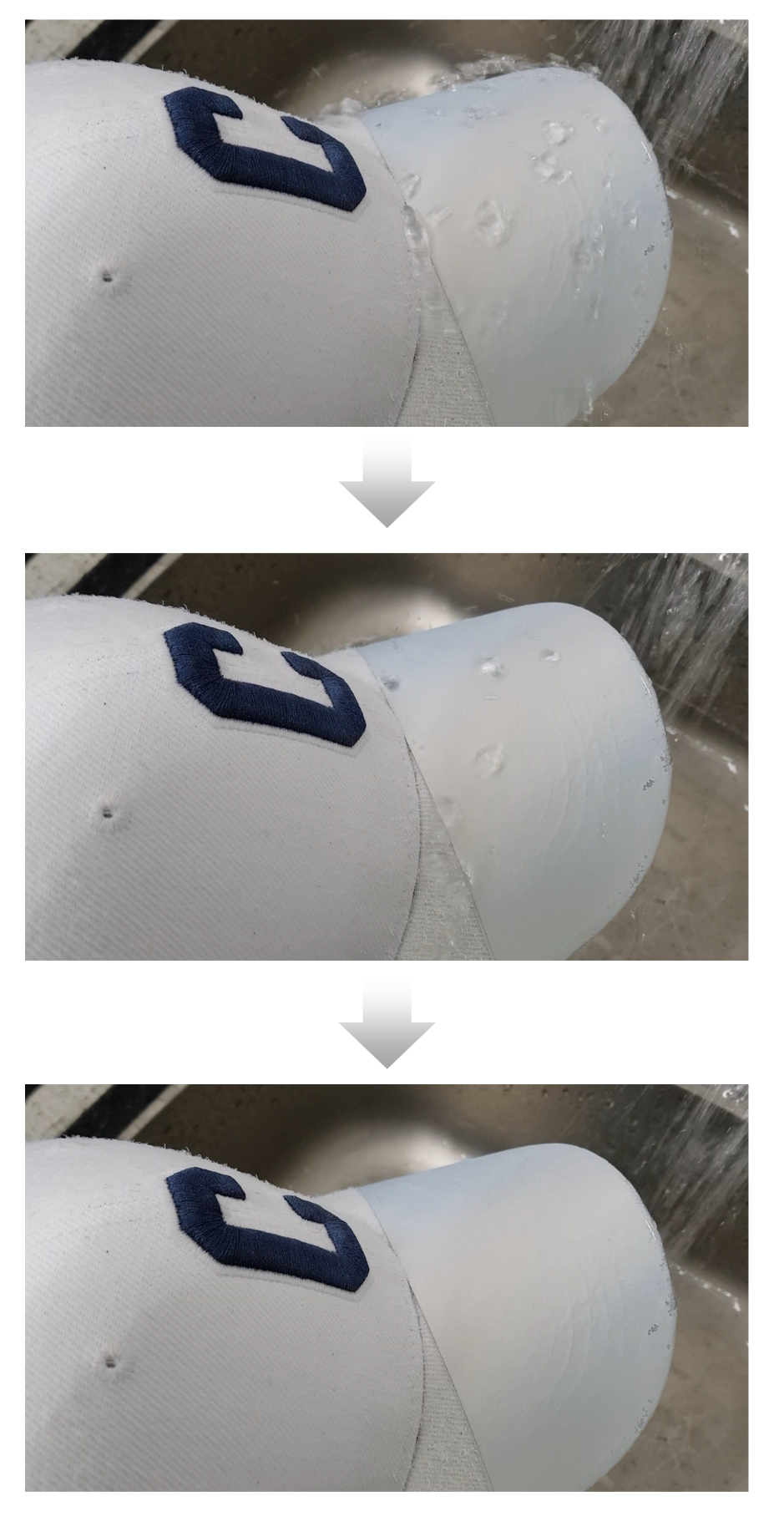


**Figure S5.** Photographs of sprayed-on TENG cap when water is applied with a commercial shower head.


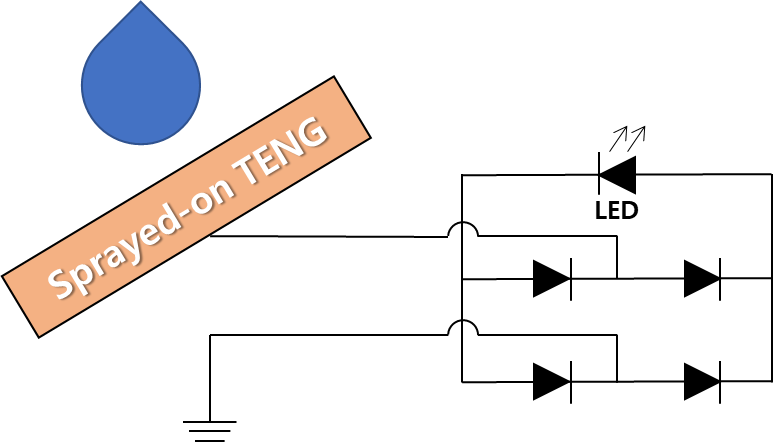


**Figure S6.** Sprayed-on TENG connected to a circuit to light an LED.
